# Supplementary material for: Genetic variation in adaptability and pleiotropy in budding yeast
Source: eLife. 2017 Aug 17;6:e27167. doi: 10.7554/eLife.27167 (PMC5580887; doi:10.7554/eLife.27167)
Supplement: Transparent reporting form [file elife-27167-transrepform.docx]

***eLife’s* transparent reporting form**

We encourage authors to provide detailed information *within their submission* to facilitate the interpretation and replication of experiments. If you have any questions, please contact us: [editorial@elifesciences.org](mailto:editorial@elifesciences.org).

**Sample-size estimation**

- You should state whether an appropriate sample size was computed when the study was being designed
- You should state the statistical method of sample size computation and any required assumptions
- If no explicit power analysis was used, you should describe how you decided what sample (replicate) size (number) to use

Please outline where this information can be found within the submission (e.g., page numbers or figure legends), or explain why this information doesn’t apply to your submission:

This is not applicable to our submission (this is an experimental evolution study on variation in adaptability, no power analysis or sample size estimation would be possible or appropriate).

**Replicates**

- You should report how often each experiment was performed
- You should include a definition of biological versus technical replication
- The data obtained should be provided and sufficient information should be provided to indicate the number of independent biological and/or technical replicates
- If you encountered any outliers, you should describe how these were handled
- Criteria for exclusion/inclusion of data should be clearly stated
- High-throughput sequence data should be uploaded before submission, with a private link for reviewers provided (these are available from both GEO and ArrayExpress)

Please outline where this information can be found within the submission (e.g., page numbers or figure legends), or explain why this information doesn’t apply to your submission:

Evolution and Fitness Data:

A description of the number of populations and biological replicates used for evolution can be found in the main text in the first paragraph of the results section, in schematic figure 1a, and in the ‘Experimental Evolution’ section of the Methods. Technical replication in the fitness measurements is described in the ‘Fitness Assays’ section of the methods. A table containing this data is in Supplemental File 2. No fitness data was excluded from the analysis.

A description of the number of populations sequenced can be found in the main text, in the ‘genetic basis of adaptation’ section, paragraph 2. A description of populations that were masked in the sequence data analysis is included in the ‘Sequencing and mutation calling’ section of the Methods. Fastq files from this project have been uploaded to the GenBank SRA (accession: SRP102877).

**Statistical reporting**

- Statistical analysis methods should be described and justified
- Raw data should be presented in figures whenever informative to do so (typically when N per group is less than 10)
- For each experiment, you should identify the statistical tests used, exact values of N, definitions of center, methods of multiple test correction, and dispersion and precision measures (e.g., mean, median, SD, SEM, confidence intervals; and, for the major substantive results, a measure of effect size (e.g., Pearson's r, Cohen's d)
- Report exact p-values wherever possible alongside the summary statistics and 95% confidence intervals. These should be reported for all key questions and not only when the p-value is less than 0.05.

Please outline where this information can be found within the submission (e.g., page numbers or figure legends), or explain why this information doesn’t apply to your submission:

Statistical data analysis methods are described in the 7 final Methods subsections, from pages 15-21. Data analysis was performed using custom Python scripts, available at <https://github.com/erjerison/adaptability>. This information is provided at the end of the first relevant methods section (p. 17).

Figure 1c contains a point for each of the final fitness measurements of a population in each environment. As noted above, this data can be found in Supplementary File 2.

The statistical tests used in the manuscript are described in the methods, p. 15-21. The main statistical results concern heritability statistics and the amount of variance explained by other models of fitness gains. These results are reported in Table 1, with 95% confidence intervals determined via a jackknife over N=230 segregants. Mutual information metrics and the permutation procedure used to calculate p-values are described in the ‘Mutual Information Analysis’ Methods section (p. 20-21) and are reported in Figure 5 - Source Data 1. The iterative procedure and permutation test used to detect QTLs, including multiple hypothesis correction, is described in the ‘Mapping QTLs’ section of the methods (p. 18). Model parameters for QTL models are reported in Figure 3 - Source Data 1 and Figure 7 - Source Data 1.

(For large datasets, or papers with a very large number of statistical tests, you may upload a single table file with tests, Ns, etc., with reference to page numbers in the manuscript.)

**Additional data files (“source data”)**

- We encourage you to upload relevant additional data files, such as numerical data that are represented as a graph in a figure, or as a summary table
- Where provided, these should be in the most useful format, and they can be uploaded as “Source data” files linked to a main figure or table
- Include model definition files including the full list of parameters used
- Include code used for data analysis (e.g., R, MatLab)
- Avoid stating that data files are “available upon request”

Please indicate the figures or tables for which source data files have been provided:

Figure 1: The fitness data for Figure 1 is in Supplementary File 2; the x-axis in Figure 1c was determined based on the genotypes in Supplementary File 1. Note that we chose not to explicitly label these files as ‘Source Data’ for Figure 1 because they are also the basis of several other analyses and contribute to other figures.

Figure 2: The source data for Figure 2 is in Table 1.

Figure 3: Fitness and genotype data used for Figure 3 in Supplementary File 2 and Supplementary File 1, respectively. Model parameters related to this figure are in Figure 3 - Source Data 1. Analysis scripts used to produce this figure can be found at: <https://github.com/erjerison/adaptability>

Figure 4: The table of mutations used for Figure 4 is in Supplementary File 3. Analysis scripts used to produce this figure can be found at: <https://github.com/erjerison/adaptability>. We chose not to explicitly link this source data to this figure because it also underlies Figure 5.

Figure 5: The table of mutations used for Figure 4 is in Supplementary File 3. Analysis scripts used to produce this figure can be found at: <https://github.com/erjerison/adaptability>. We chose not to explicitly link this source data to this figure because it also underlies Figure 4. Mutual information statistics related to further analysis of this data, and discussed in the main text on p. are found in Figure 5 - Source Data 1.

Figure 6: The fitness data used in this figure is contained in Supplementary File 2. Analysis scripts used to produce this figure can be found at: <https://github.com/erjerison/adaptability>.

Figure 7: The source data for Fig. 7a. and Fig. 7b. is in the final two columns of Table 1. The fitness data used in Fig. 7c. and Fig. 7d. is contained in Supplementary File 2. Model parameters for Fig. 7c. and Fig. 7d. can be found in Figure 7 - Source Data 1.

As noted above, analysis scripts can be found at: <https://github.com/erjerison/adaptability>
